# Smart interfaces to assist the operator in the context of industry 4.0 with a 5S human-centric approach

**DOI:** 10.1017/wtc.2024.17

**Published:** 2024-12-23

**Authors:** Mario Rojas, Javier Maldonado-Romo, Juana Isabel Mendez, Pedro Ponce, Arturo Molina

**Affiliations:** Institute of Advanced Materials for Sustainable Manufacturing, Tecnologico de Monterrey, Monterrey 64849, Nuevo Leon, Mexico

**Keywords:** haptic-gloves, virtual-reality, smart-interfaces, human-centric, SDGs

## Abstract

This paper explores the integration of haptic gloves and virtual reality (VR) environments to enhance industrial training and operational efficiency within the framework of Industry 4.0 and Industry 5.0. It examines the alignment of these technologies with the Sustainable Development Goals (SDGs), mainly focusing on SDG 8 (Decent Work and Economic Growth) and SDG 9 (Industry, Innovation, and Infrastructure). By incorporating a human-centric approach, the study leverages haptic gloves to provide realistic feedback and immersive experiences in virtual training environments. The gloves enable intuitive interaction, enhancing the training efficacy and reducing real-world operational errors. Using the 5S principles—Social, Sustainable, Sensing, Smart, and Safe—this research evaluates the system’s impact across various dimensions. The findings indicate significant improvements in user comfort, productivity, and overall well-being, alongside enhanced sustainability and operational efficiency. However, challenges related to realistic hand-object interactions and algorithmic enhancements were identified. The study underscores the importance of continuous improvement and cross-disciplinary collaboration to advance the usability and effectiveness of these technologies. Future research should focus on customization, AI-driven adaptability, sustainability, real-world scalability, and comprehensive impact assessment to further develop smart interfaces in industrial settings. This integration represents a transformative opportunity to enhance workplace safety, skills development, and contribute to global sustainable development goals.

## 1. Introduction

Environmental protection, resource efficiency, and social well-being are now integral components of “green growth,” a sustainable development approach adopted by governments and industries worldwide. These policies are closely aligned with the 17 Sustainable Development Goals (SDGs) set by the United Nations in 2015 to guide global development efforts until 2030 (UN DESA, 2023). The SDGs focus on achieving sustainable development across environmental, economic, and social dimensions. The social dimension has recently gained recognition for its importance in ensuring that economic growth and environmental progress benefits are shared inclusively. Social inclusion efforts emphasize job creation, poverty reduction, access to essential services, and social protection policies. Within the industrial sector, two SDGs focus on workers’ rights, well-being, and economic empowerment: SDG 8 (Decent Work and Economic Growth) and SDG 9 (Industry, Innovation, and Infrastructure). SDG 8 prioritizes sustained, inclusive, and sustainable economic growth, full employment, and decent work opportunities for all, including industrial workers. SDG 9 aims to enhance resilient infrastructure, foster inclusive and sustainable industrialization, and drive innovation, directly benefiting industry workers (Machado et al., 2019).

Anthropocentrism, driven by Industry 4.0, is crucial in shaping future production within smart factories (Rauch et al., 2020). This human-centric perspective emphasizes design, empowerment, ethics, sustainability, and social responsibility. Smart factories can achieve heightened efficiency, efficacy, and societal benefits by prioritizing human elements in technological innovation. Therefore, anthropocentrism advocates for empowering workers and fostering engagement while cultivating a culture of continuous learning and skill development to keep pace with technological advancements. Responding to emerging societal trends, the European Commission proposed Industry 5.0 (European Commission et al., 2020), focusing on human-centricity, ecological benefits, and social benefits rather than the techno-centric perspective of Industry 4.0. In smart factories, this entails providing workers with tools and technologies that enhance their skills and enable meaningful contributions to production processes.

Researchers have identified the need for development to support the transition from technocentric Industry 4.0 to value-centered Industry 5.0. Technologies based on ethical considerations that support human values and needs are necessary (Enang et al., 2023). The rise of novel interaction techniques for smart products, machines, and operators requires reevaluating human-machine relationships (Xu et al., 2021). Additionally, empowering workers with digital technologies in this new era can accelerate progress toward the SDGs.

### 1.1. Digital transformation and emerging technologies

Digital transformation is key to boosting industry competitiveness, especially in emerging countries. It impacts organizations, business models, and customer experiences by driving change and innovation. This transformation involves restructuring and cultural changes to leverage digital technologies for new business models (Nadkarni & Prügl, 2020). Technologies such as the Internet of Things (IoT) enhance customer relationships with products and services. Big Data Analytics supports data-driven decisions, providing a competitive advantage. Cloud computing fosters innovation, while Artificial Intelligence (AI) and Machine Learning (ML) automate processes and improve decision-making. Blockchain and cybersecurity ensure data protection, and Robotic Process Automation (RPA) reduces workloads and prevents accidents (Ponce et al., 2023).

Digital technologies aim at Industry 4.0, creating smart, connected, and automated systems in the manufacturing and industrial sectors. A key component is virtual reality (VR) technology (Frank et al., 2019). VR generates immersive, simulated environments for user interaction. These systems employ specialized hardware and software to immerse users in virtual environments, simulating decision-making processes in authentic factory settings (Gorecky et al., 2017). A proposed framework by Ponce et al. (2024) uses VR for learning advanced manufacturing and enhancing skills with realistic simulations. AI-driven training adapts to individual needs, optimizing processes, reducing waste, and increasing productivity. VR supports virtual prototyping and testing, speeds up product development, and enables global collaboration in advanced manufacturing.

VR offers interactive educational experiences resembling real-world conditions, serving as a dynamic alternative to traditional training methods (Holuša et al., 2023). It enables skill enhancement without risking damage to real-world objects or exposure to hazards before adequate training (de Assis Dornelles et al., 2022). Research studies reveal growing enthusiasm for VR training across different industries, demonstrating its efficacy in transferring procedural, decision-making, spatial, and fine/gross motor skills (Radhakrishnan et al., 2021). Additionally, methodologies for integrating VR into Industry 4.0 education highlight the merits and considerations of VR-based education (Paszkiewicz et al., 2021). VR is positioned within digital factories, enhancing training and maintenance systems. VR headsets and handheld devices offer step-by-step instructions to guide assembly and maintenance operators during training (Adattila et al., 2024). Transitioning from paper-based to multimedia instructions with AR and VR enhances operator cognitive abilities and decision-making skills and reduces cognitive workload (Adriana Cárdenas-Robledo et al., 2022).

Smart interfaces complement the user’s interactive experiences with realistic feedback, enhancing training effectiveness and improving learning outcomes for skill acquisition. For instance, smart gloves like Manus Prime II, as introduced by Manuri et al. (2023), are used with headset controllers in immersive VR applications for rapid, low-cost automation prototyping. These haptic gloves, equipped with sensors and actuators, enable users to interact with virtual environments through gestures and tactile feedback. Wearable haptic interfaces facilitate communication and cooperation between humans and machines, offering natural and private interaction with the shared environment (Pacchierotti et al., 2017). Advancements in flexible sensors and wireless electronics have led to the development of lightweight, ergonomic, wearable sensing gloves for mixed reality (MR) applications. Kim et al. (2023) incorporated a smart gloves prototype with integrated capacitive pressure sensors for haptic feedback during object interaction, highlighting quantitative assessments of users’ interactions. Additionally, wearable devices and environmental sensors enhance AR training by collecting worker data to improve training experiences (de Assis Dornelles et al., 2022). Luo et al. (2024) presented a textile-based wearable human-machine interface embedding tactile sensors and vibrotactile haptic actuators into textiles, enabling smart gloves to acquire users’ data for investigating their perception. Zhu et al. (2020) proposed a haptic-feedback smart glove with finger bending sensors, palm sliding sensors, and piezoelectric mechanical stimulators for detecting bending and sliding events in a virtual space, augmenting human-machine interaction.

### 1.2. 5S human-centric approach

The interaction between humans and machines, spanning digital and physical realms, represents a crucial link shaped by ongoing socio-technical evolution. As discussed by Mark et al. (2021), manufacturing worker assistance systems promise to enhance employee well-being and company performance. Smart interfaces and augmented technologies enhance efficiency, safety, and training, enabling data-driven decision-making in smart factory environments. Moreover, the transition from Industry 4.0 to Industry 5.0 is not just a transition but a call to action. It involves developing human-centric technological approaches for healthier, safer, and more productive workforces and environments. This urgent need for change aims to realize the socially sustainable factories of the future (Adattila et al., 2024). Thus, Industry 5.0 is not merely a technology-driven revolution but a value-driven initiative, necessitating personalized human-machine interaction technologies to integrate and leverage the strengths of humans and machines (Müller et al., 2020).

The field of smart interfaces, including emerging technologies like haptic gloves, is advancing despite current limitations and challenges. Countries can collaborate by sharing knowledge, best practices, and expertise in developing and implementing smart and green technologies. Capacity-building programs transfer skills and build local capabilities, while joint research initiatives pool resources to innovate sustainable solutions swiftly. Harmonizing policies and regulations globally supports the widespread adoption of these technologies. International financial support accelerates the implementation of sustainable projects, and public-private partnerships leverage diverse expertise to achieve shared development goals. Facilitating technology transfer programs ensures rapid adoption of innovative solutions worldwide, driving economic growth, job creation, and improving quality of life while contributing to multiple SDGs (Mondejar et al., 2021).

Despite these advancements, the usability of such digital technologies in practical, real-world settings is underexplored. Usability, defined as the extent to which a system can be used by specified users to achieve specified goals with effectiveness, efficiency, and satisfaction, is crucial for the adoption and success of these technologies. The concept of Cellulographics, introduced by Kalia et al. (2022), offers a comprehensive behavioral classification based on various user characteristics such as smartphone experience, skill, internet experience, usage periods, screen time, usage frequency, activities, and location. This framework provides valuable insights into user behavior and preferences, critical for designing user-centric digital interfaces in industrial applications. Furthermore, Cellulographics can be adapted to haptic interfaces such as gloves and VR usage. The framework can help design more intuitive and effective haptic feedback systems by understanding user interactions with these technologies. For instance, the classification metrics can be used to tailor haptic responses based on the user’s experience and skill level, ensuring a more personalized and engaging VR experience. This adaptation enhances the usability and effectiveness of haptic interfaces, making them more accessible and beneficial for a wider range of users.

Additionally, as proposed by Nielsen, usability heuristics provide a robust framework for evaluating the usability of these digital technologies (Capeleti et al., 2020). These heuristics emphasize the importance of visibility of system status, ensuring that users are informed about what is happening through appropriate feedback; the match between the system and the real world, using language and concepts familiar to users; user control and freedom, allowing users to undo and redo actions; consistency and standards to avoid confusion; error prevention; recognition rather than recall to minimize memory load; flexibility and efficiency of use, accommodating both novice and expert users; aesthetic and minimalist design; help users recognize, diagnose, and recover from errors through clear error messages; and providing help and documentation to support user needs.

To benefit workers and achieve the SDGs, efforts to utilize digital transformation and cutting-edge technologies need to integrate the operator as a central entity. Concepts like Industry 5.0 recognize the importance of the operator but require further evidence and implementation. It is crucial to prioritize operators and make them the primary focus when developing technology for the industry to enhance sustainability. This approach leverages emerging technologies that can reduce costs through effective implementation. Han et al. (2021) discusses the importance of measuring sustainable product design concepts to minimize adverse environmental impacts. The study highlights three knowledge gaps in current design practices: the need for more consideration of environmental impact, challenges in improving sustainability post-design, and the inadequacy of existing concept assessment methods in ensuring sustainable design. It proposes four metrics (material, production, use, and end of life) for evaluating sustainable product design concepts. A case study on portable blender concepts demonstrates the application of these metrics, emphasizing the significance of addressing sustainability issues at the conceptual design stage to achieve more sustainable products.

Given the SDGs requirements, proposing solutions that promote decent work and economic development is imperative. However, the disparity in resource availability across different regions presents a challenge in meeting these objectives in the short term. Consequently, advocating for solutions grounded in sustainable technological advancements is essential. This study employs five dimensions, encompassing core aspects of sustainability: economic, social, and environmental factors, and introduces two supplementary dimensions: circularity and technological development.

- Kretschmar et al. (2023) discusses the importance of sustainability in corporate activities and how product-service systems (PSS) can be a sustainable opportunity through efficient resource use and value generation. The study identifies sustainability indicators in the automotive sector and develops a business model approach that integrates sustainability aspects and PSS requirements. The research approach involves selecting a suitable framework for corporate sustainability, designing a business model approach focusing on sustainability indicators and PSS requirements, and combining them in an impact model. The paper emphasizes the need for a holistic view of sustainability, considering ecological, economic, and social aspects.
- Calik and Bardudeen (2016) discusses the development of a measurement scale to evaluate sustainable innovation performance in manufacturing organizations. It emphasizes the importance of considering sustainability’s economic, environmental, and societal aspects in measuring innovation. The study reviews existing literature on sustainable innovation measurement and highlights the need for a comprehensive scale incorporating these dimensions. The proposed measurement model focuses on result and output-oriented processes, particularly at the product and process levels.
- The study classifies sustainability assessment into product and process categories, outlining various methodologies, advantages, and weaknesses. Key issues identified include the need for comprehensive assessment covering environmental, social, and economic aspects, limitations in analyzing qualitative data, and boundary constraints in some methodologies. The review aims to address gaps in the existing literature and offers insights for future research in sustainable product and process development (Longo et al., 2021).

Therefore, to measure the impact of these technologies comprehensively, Molina et al. (2024) proposes the 5S principles: Social, Sustainable, Sensing, Smart, and Safe. These principles ensure that the development and implementation of technologies are efficient, innovative, responsible, and mindful of broader societal and environmental impacts. Thus, the 5S principles can be described as:

- Social: Ensuring ethical practices, inclusivity, and positive community engagement. This includes enhancing employee comfort, productivity, and overall well-being and integrating usability testing and user-centered design principles to ensure intuitive products.
- Sustainable: Minimizing environmental footprints and promoting resource conservation. This involves reducing the use of harmful raw materials in manufacturing, reducing greenhouse gas emissions, designing products that minimize resource consumption and pollution, reducing energy consumption, optimizing material usage, promoting recycled materials and reused components, and enhancing the efficiency of recycling processes.
- Sensing: Integrating sensors and data collection technologies to gather insights and enhance decision-making. This includes considering low-resource investments for employing the interface and prioritizing proactive maintenance practices to extend the lifespan of equipment and infrastructure.
- Smart: Incorporating advanced technologies to create adaptive and intelligent systems. This involves designing products to maximize utility during their use phase and optimizing the performance of assistance to workers.
- Safe: Prioritizing safety and security for individuals, communities, and data integrity. This includes ensuring high-quality standards, maximizing durability, improving workplace health and safety, and establishing rigorous quality assurance processes.

### 1.3. Aims and objectives

This study aims to bridge the gap between traditional industrial practices and advanced digital training methodologies. By focusing on human-machine interaction, particularly through VR and haptic technologies, this research seeks to enhance workers’ training and operational efficiency in industrial settings.

- Demonstrate the efficacy of virtual reality VR environments combined with haptic feedback gloves in enhancing the training experience for workers, particularly in the context of operating industrial machinery such as milling machines. This aims to provide a safe, interactive, and immersive learning environment that reduces the risk of accidents and significantly improves skill acquisition.
- Evaluate the transition from traditional training methods to a technologically enhanced learning approach using VR and haptic gloves. This includes assessing how well skills learned in a virtual environment translate to real-world settings, directly impacting operational safety and efficiency.
- Adopt a human-centric approach to technology implementation in industrial environments, ensuring that the development and application of technologies are socially sustainable, promote safety, and are user-friendly. This approach is encapsulated in the 5S principles—Social, Sustainable, Sensing, Smart, and Safe—guiding the evaluation of various dimensions of technology impacts.

The remainder of the manuscript is presented as follows. Section 2 reviews the materials and methods. Likewise, the experiment and the analysis of the results and discussion are detailed in Sections 3 and 4, respectively. Finally, Section 5 presents conclusions and future work.

## 2. Materials and methods

By nature, humans are adept at manipulating objects with their hands, rendering interactions intuitive and with minimal training. Thus, a haptic glove is proposed to serve as an interface between the virtual and physical environments through haptic feedback. The glove elevates user immersion by offering feedback through motor vibrations and validating interactions within virtual settings. Its use fosters a more intuitive and immersive experience compared to traditional controller-based methods.

This interface will be integrated into a virtual training environment to familiarize workers with machinery operations before using it in a real-world setting. This training program aims to reduce accidents caused by inexperienced manufacturing equipment handling. The prototype development involves five key stages:

1. Electronic circuit design
2. The haptic glove assembly
3. The virtual environment design
4. The system’s hardware and software integration
5. Evaluation of the prototype

**Figure 1.**
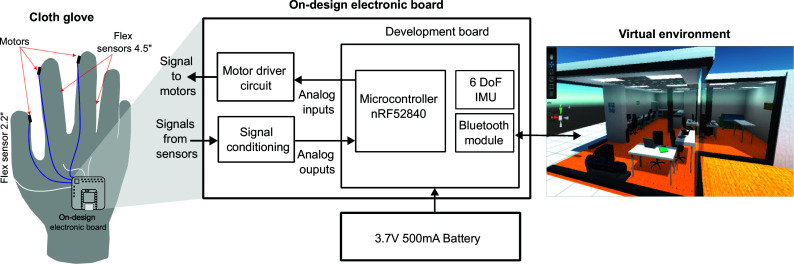
The general block diagram of the system.

Figure 1 provides a schematic overview of the system’s components, highlighting three primary blocks: the cloth glove, the custom electronic board, and the virtual reality environment. The cloth glove utilizes two 4.5″ flex resistive sensors to detect bending movements in the index and middle fingers and a 2.2″ sensor for the thumb. Additionally, three compact vibrating DC motors provide haptic feedback to these fingers. As shown in the glove diagram in Figure 1, the sensors’ signals are wired directly to the microcontroller’s analog inputs, while the DC motors connect to PWM outputs. The custom PCB on the electronic board includes signal conditioning circuits for the sensors, motor-driving circuits, and a Seeed Studio XIAO development board. A 3.7 V lithium polymer battery powers the microcontroller and peripherals, and data is transmitted to the computer running the virtual scenario via Bluetooth. The scenario features detailed 3D models of machinery, interactive controls, and guided instructions, allowing trainees to practice and gain proficiency before transitioning to real-world equipment. This virtual training ensures operators are well-prepared to operate the equipment safely and efficiently. A detailed description of these blocks is provided in the following sections.

### 2.1. Electronic design

**Figure 2.**
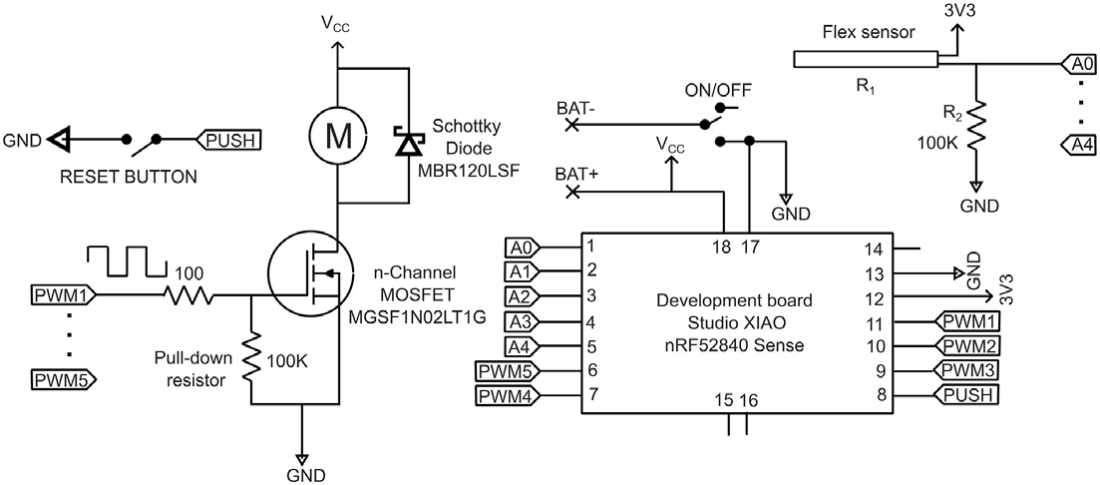
The electric components diagram.

A circuit was designed to incorporate the electronic components for both the signal conditioning and the motor driver functions. Also, signals from the flex sensors were routed to the microcontroller’s digital inputs, while PWM output signals were directed to the motor drive circuit to power the motors. This design considered the inclusion of resistive sensors and motors in each of the five glove fingers as illustrated by the diagram in Figure 2.

#### 2.1.1. The microcontroller description

The Seeed Studio XIAO nRF52840 (Sense) development board is compact, measuring less than 2.5 cm (0.98 in) in height and width. It features a Nordic nRF52840 microcontroller unit with Bluetooth 5.0 connectivity, a built-in Bluetooth antenna, a 6 Degree-Of-Freedom Inertial Movement Unit (IMU), a PDM microphone, an RBG LED, and a TI BQ25101 charger chip with a charge LED indicator. Additionally, it includes a reset button, 11 PWM pins, 6 analog pins, and support for popular communication protocols. With a USB-C connector and single-sided surface-mountable design for programming, the board operates on a 3.3 V reference voltage, eliminating the need for a buck or boost converter for compatibility with sensors, indicators, and actuators in the prototype.

#### 2.1.2. The flex sensors signal conditioning

As finger flexion increases from an extension position, the sensors’ resistance changes according to the diagram presented in the curve of the bending angle versus the sensor’s resistance presented in (Al-Rahayfeh & Faezipour, 2014). The effectiveness of flex sensors for recognizing finger gestures is also highlighted in (Chuang et al., 2019).

Voltage divider circuits were utilized to derive a proportional signal from the flex sensors, which adjust their resistance according the finger flexion. Fixed-value resistors were used for each sensor within the voltage divider setup to measure resistance changes, enabling the conversion of the flex sensors’ output voltage into quantifiable values. Thus, the output voltage is determined by the following relation:
(2.1)

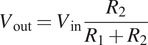



**Figure 3.**
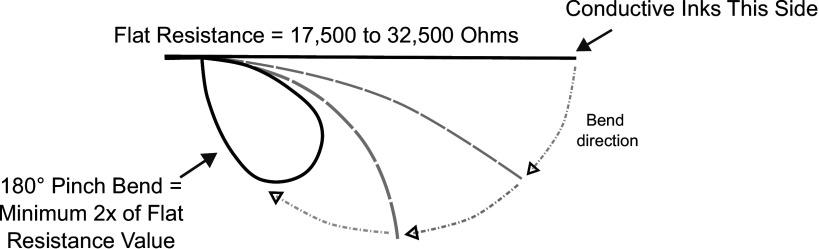
The bending diagram obtained from (flex_sensor).

Where 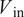 represents the reference voltage, with 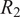 denoting a fixed-value resistor, and 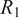 symbolizing the variable resistance provided by the flex sensor. The minimum resistance of 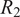 is twice the flat resistance value at a 180° pinch bend, as depicted in Figure 3. Based on the sensors’ specifications, 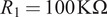 was calculated for the 2.2″ flex sensor, while 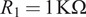 was selected for the 4.5″ flex sensors.

#### 2.1.3. The driver circuit for the vibrating motors

A driver circuit is needed to regulate power flow for the 8 mm diameter vibrating motors, chosen for their suitability in haptic applications, as shown by Li et al. (2020). These motors can be powered by voltages ranging from 3 V to 5 V and draw currents between 74 mA and 130 mA. Placed directly beneath the fingertips, they provide subtle vibrations for the desired haptic feedback.

The vibration motors require 84 mA at 3.7 V, exceeding the current typically provided by microcontroller ports. To address this, a common solution involves utilizing enhancement-type MOSFETs, known for their efficiency and robustness. The circuit configuration, depicted in Figure 2, comprises an n-channel MOSFET, a 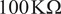 pull-down resistor, a 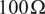 gate resistor, a 100 pF ceramic capacitor, and a flyback Schottky diode.

#### 2.1.4. The PCB integration

A customized PCB was designed to incorporate all the electronic components depicted in Figure 2. Employing Surface Mount Device (SMD) technology ensured the integration of smaller and lighter components compared to traditional through-hole counterparts. This not only enabled a reduction in board size but also enhanced mechanical stability, given that SMD components are soldered directly onto the PCB surface, thereby ensuring resistance against vibration and shock.

### 2.2. The haptic glove design and assembly

**Figure 4.**
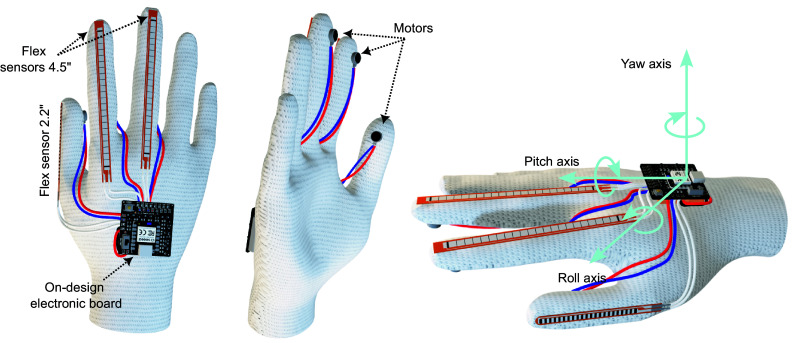
Design of the glove with assembled components and the orientation angles.

Figure 4 displays a 3D rendering of the gloves showcasing the components from both front and rear perspectives. Three flex sensors are affixed to the thumb, index finger, and middle finger of the glove, while vibration motors are positioned on the fingertips. Actuators and sensors are interconnected to the PCB via copper wires, with the battery securely fastened to the flat side of the PCB, which is firmly attached to the glove. Also, Figure 4 shows the orientation angles for the glove. Using hand movements as an interface for interacting with the virtual environment can achieve a more intuitive and immersive experience than traditional controller-based interactions. The IMU sensors provide information about the hand’s linear acceleration and angular velocity. This data is used as input for mathematical algorithms and formulas that allow for the estimation of Euler angles. Euler angles, such as roll ( 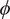 ), pitch ( 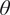 ), and yaw ( 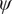 ), are common representations used to describe the orientation of an object in real-time.

The formulas to determine the orientation are described next:
(2.2)

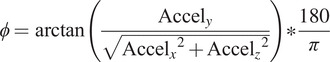



(2.3)

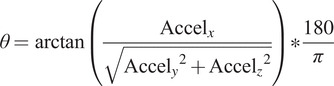



(2.4)

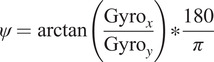

where 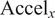 , 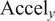 , 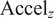 , 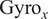 , 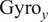 are the linear acceleration and angular velocities of the object, respectively.

**Figure 5.**
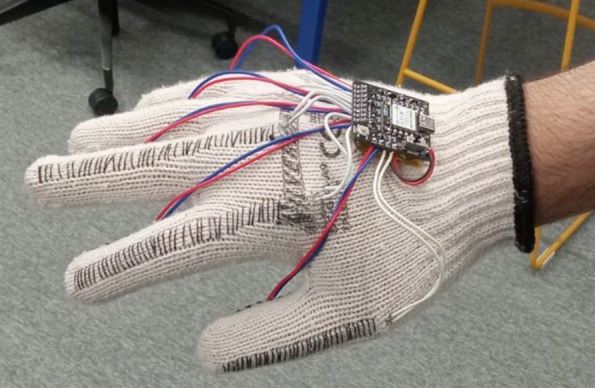
Final prototype featuring all the components.

Figure 5 shows a user wearing the final prototype with the installed components. The selected glove, the Silverline DC-GUA-A9 Cotton Knitted Glove with PVC Dots provides an enhanced grip for handling objects and tools. Made of 100% cotton yarn and jacquard fabric, it is comfortable, lightweight, breathable, and flexible, suitable for various conditions. This choice aligns well with industry standards, as similar gloves are commonly worn by workers in the field.

### 2.3. The virtual environment design

The virtual environment design was inspired by a real laboratory located at the Mexico City campus of Tecnologico de Monterrey, a private university. Utilizing a smartphone, specifically an iPhone 12 Pro equipped with a LiDAR (Light Detection and Ranging) sensor, a 3D scan of the laboratory was captured. This scan served as the foundation for the virtual environment creation process within Unity, a versatile cross-platform game development engine and software development kit (SDK). Unity facilitates the development of interactive experiences ranging from video games to simulations and various applications.

**Figure 6.**
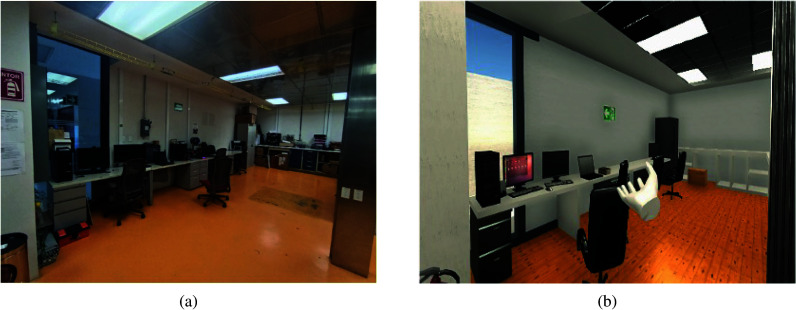
Comparison between the virtual and real world scenarios. (a) Real. (b) Virtual.

In addition, supplementary elements, including furniture, materials, textures, and lighting sources, were integrated to elevate realism within the virtual environment. Utilizing photographs of the actual space, textures were chosen to represent colors and other details accurately. Moreover, the virtual environment was meticulously configured to emulate physical attributes of objects, encompassing collisions, basic movement, and additional interactions, thereby enhancing the overall immersive experience. A comparison between the real facilities and the final virtual scenario is shown in Figure 6.

#### 2.3.1. The virtual hand model

**Figure 7.**
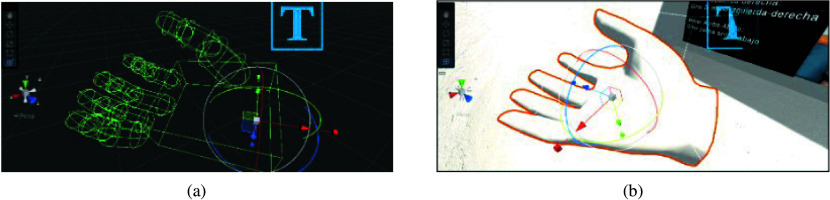
Details of the virtual glove model. (a) Articulations. (b) Object with texture.

A 3D hand model used in VR applications was downloaded from (MetaQuest, 2017) for tracking the user’s movements captured by the glove. This model features an articulation component in each movable part, closely resembling a real hand, as presented in Figure 7. Subsequently, an animation script for representing specific gestures was created in response to the real-world flexion of the sensors and hand rotation. Those scripts share information with the haptic glove prototype through a serial port configuration.

### 2.4. Hardware and software integration

#### 2.4.1. The training tool: starting a milling machine

A milling machine is a versatile tool that supports teaching and research activities in a university laboratory. University laboratories often need custom-made parts for equipment or research setups, and a milling machine allows them to fabricate these parts with precision and accuracy.

**Figure 8.**
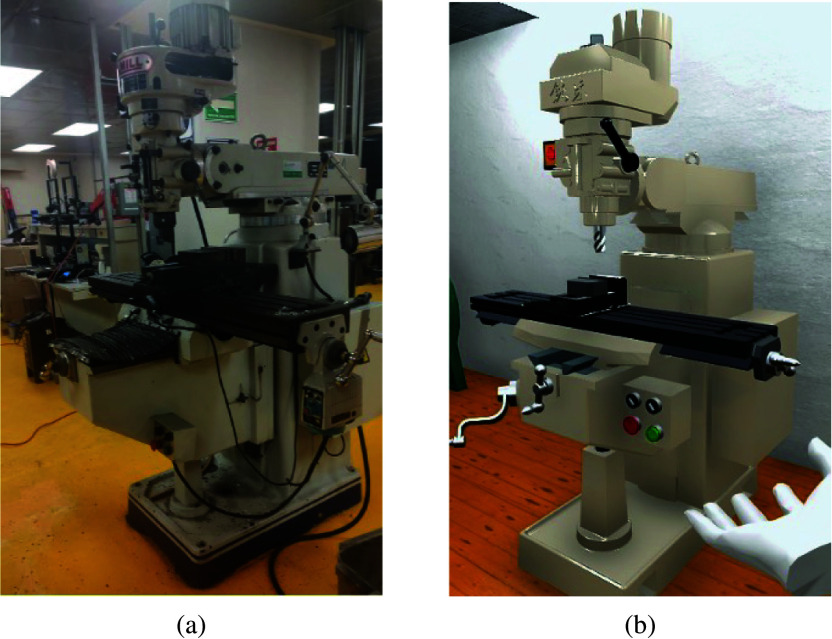
The milling machine for the proposed training activity. (a) Real-world machine. (b) Virtual model.

Using a milling machine in a university laboratory can be hazardous due to several factors. Firstly, the rotating cutting tool poses a risk of entanglement or contact injuries if not handled properly. Additionally, the sharp cutting tools and generated chips can cause cuts, punctures, or eye injuries if safety precautions are not followed. Moreover, the noise, vibrations, and potential electrical hazards associated with milling machines further increase the risk of harm. A virtual model of a laboratory machine was included in the virtual environment, as shown by Figure 8.

Before using a real-world milling machine, students should ensure to receive proper training on safe operation, wear appropriate personal protective equipment, inspect the machine for any defects, secure the workpiece properly, and follow all safety procedures outlined by the laboratory supervisor or instructor. These considerations are crucial for minimizing the risks and ensuring a safe working environment for the students.

#### 2.4.2. The user’s evaluation of the training tool

**Figure 9.**
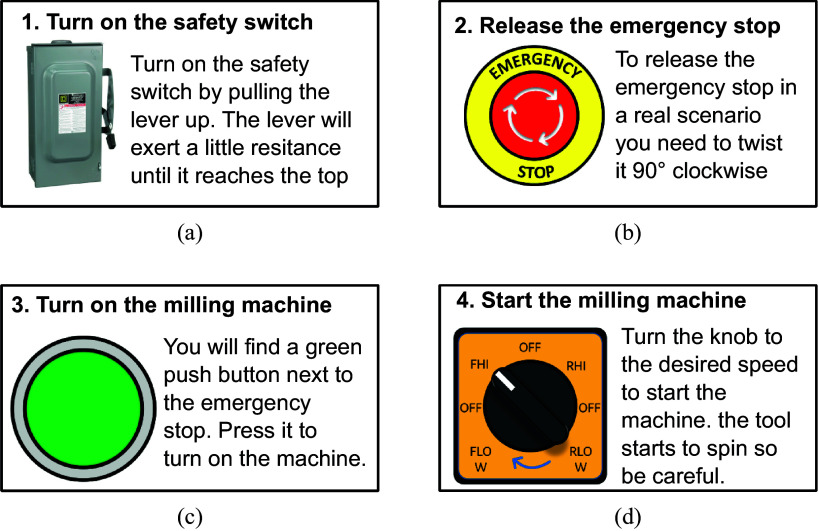
The training steps for starting the milling machine.

The proposed virtual environment included a step-by-step tutorial on how to operate the machinery. The haptic glove prototype underwent validation tests with users to complete a training activity. According to (Experience, 2000), the best results come from testing no more than 5 users thus five students from the campus participated in the evaluation. The goal was to assess the effectiveness of the system in transferring knowledge and skills to real-world scenarios. The users were trained in the virtual environment to perform the specific steps for starting a milling machine, which are shown in Figure 9. After the virtual training, they tested the skills they had learned on the real milling machine.

The evaluation process used a questionnaire to gather feedback on the training experience, considering the usability aspects: ease of learning, efficiency, memorability, errors, and satisfaction. The proposed questions were organized as “before”, “during”, and “after” the training activity as shown in Table 1.

**Table 1.** Questions to measure the impact from the users

*Stage: Before virtual training*
Question 1: Do you have previous experience using haptic gloves?
Question 2: Do you have previous experience in the manufacturing environment?
Question 3: What types of milling machines are you familiar with?
*Stage: During virtual training*
Question 4: After performing the milling machine startup routine in the virtual environment, how many mistakes did you make?
Question 5: Did the haptic glove fulfill its function and allow you to interact properly with the virtual environment?
Question 6: Do you consider the virtual environment to be intuitive and easy to understand?
*Stage: After virtual training*
Question 7: After completing the real milling machine startup routine, how many mistakes did you make?
Question 8: How would you rate the effectiveness of the training system in transferring knowledge from the training to real-world application?
Question 9: Did the training system in the virtual environment help you reduce errors when using the real milling machine?

## 3. Results

**Figure 10.**
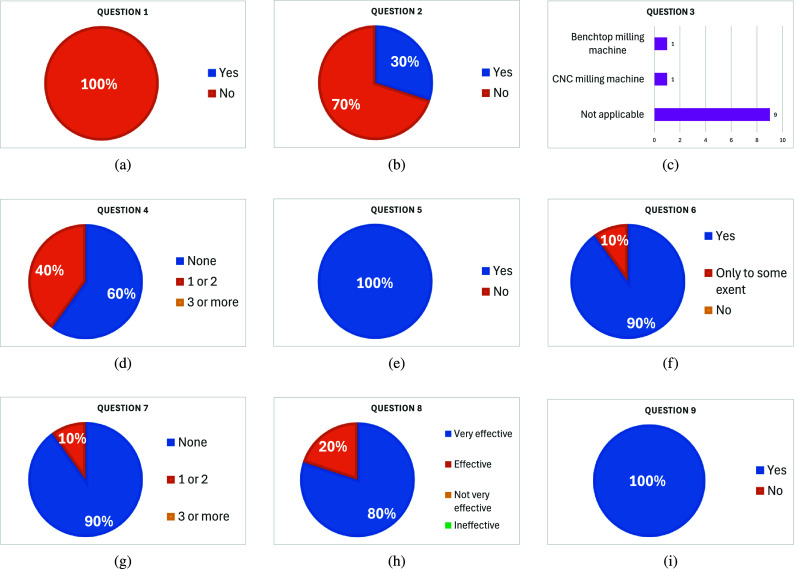
Responses obtained from the users evaluation.

The questionnaire results provided valuable insights into the effectiveness of the user experience and the training system. Questions 1, 2, and 3 corresponded to the before virtual training stage and offered valuable insights for enhancing learning outcomes. According to the responses shown in Figure 10(a), (b), (c), the participants exhibited diverse levels of experience and knowledge. None had prior exposure to haptic gloves, but all were familiar with virtual reality. Additionally, while most lacked prior experience in manufacturing environments, their knowledge of milling machines varied.

The responses for questions 4, 5, and 6 are shown in Figure 10(d), (e), (f). These questions focused on the stage during virtual training, helping identify technical and instructional gaps, enabling timely adjustments to improve training effectiveness and engagement. Most users encountered 1–2 errors during the routine, with Step 2 being the most confusing. However, overall, the majority of participants found the virtual environment intuitive and easy to comprehend. The haptic glove’s ergonomics received generally positive ratings, ranging from good to excellent, enabling users to interact effectively with the virtual environment. The addition of haptic sensation to the glove contributed significantly to the training experience, adding value for the users.

In the final stage of the questionnaire, questions 7, 8, and 9 aimed to gather critical insights into the training’s real-world effectiveness, skill transferability, and to identify gaps between virtual and real-world applications. The results for this stage are shown in Figure 10(g), (h), (i). Users reported that the virtual training significantly helped reduce errors when operating the actual milling machine. They rated various aspects, including effectiveness, precision, intuitiveness, and satisfaction, highly. Additionally, learning ease, efficiency, memorability, and ergonomics received positive evaluations. Participants unanimously agreed on the necessity of a complete haptic glove for a similar experience, although they acknowledged that alternative devices could be viable considerations.

**Figure 11.**
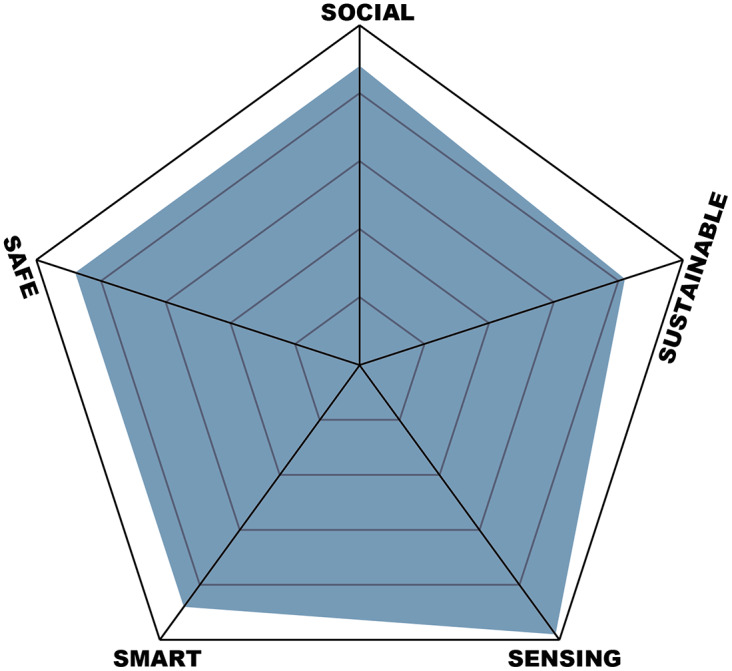
5S principles impact analysis.

Based on a 5S Human-Centric approach, the 5S principles proposed by (Molina et al., 2024) were used in this proposal. Each dimension is composed of a cluster of elements. Table 2 describes the factors and the sentences to determine a value and its association with each S principle (Social, Sustainable, Sensing, Smart, Safe). The value comprises the normalized factors using a Likert scale (Sullivan & Artino, 2013), as shown in Figure 11.

**Table 2.** Factors for each S principle

5S Principle	Factor	Question	Value
Social	Ergonomic	Does it enhance employee comfort, productivity, and overall well-being?	0.9
Social	Usability	How can usability testing and user-centered design principles be integrated into product development processes to ensure that products are intuitive?	0.85
		***Average***	***0.88***
Sustainable	Reduce the use of harmful raw materials	Does it reduce the dependency on harmful raw materials in manufacturing processes?	0.7
Sustainable	Reduce greenhouse gas emissions	How can it sustainable the system reduces greenhouse gas emissions?	0.9
Sustainable	Green product design	Does it minimize resource consumption and pollution throughout their lifecycle?	0.85
Sustainable	Reduce energy use	Does it reduce energy consumption?	0.85
Sustainable	Material usage	Does it optimize material usage through efficient inventory management?	0.9
Sustainable	Recycled materials and reused components	Does it promote the widespread adoption of recycled materials and reused components?	0.85
Sustainable	Efficiency of recycling	Does it enhance the efficiency of recycling processes, including collection, sorting, and processing, to maximize resource recovery and minimize waste?	0.7
		***Average***	***0.82***
Sensing	Infrastructure investment	Does it consider a low investment of resources to employ the interface?	1
Sensing	Maintenance	Does it prioritize proactive maintenance practices that extend the lifespan of equipment and infrastructure?	0.95
		***Average***	***0.98***
Smart	Utility during use phase	How can products be designed and optimized to maximize utility during their use phase?	0.9
Smart	Innovation	What is considered the level of innovation?	0.9
Smart	Performance	Does it optimize the performance of the assistance to workers?	0.85
		***Average***	***0.88***
Safe	Quality and durability	Does it ensure high-quality standards and maximize durability?	0.9
Safe	Health & safety	How can workplace health and safety be effectively improved to ensure the well-being of employees?	0.9
Safe	Quality assurance	Does it establish rigorous quality assurance processes that uphold product standards and customer satisfaction?	0.85
		***Average***	***0.88***

During the training, identifying technical and instructional gaps facilitated timely enhancements, making the virtual environment more intuitive and the haptic gloves more effective. The training’s success in reducing real-world operational errors underscores its practical value. Additionally, integrating the 5S Human-Centric approach ensured a holistic evaluation across various dimensions—social, sustainable, sensing, smart, and safe.

- The Social dimension scores highly, with an average of 0.88, reflecting the system’s positive impact on employee comfort, productivity, and overall well-being. The ergonomic factor received a score of 0.9, indicating that the system significantly enhances user comfort and productivity, contributing positively to their overall well-being. Usability scored 0.85, suggesting that the system is intuitive and user-friendly, thanks to effective usability testing and the integration of user-centered design principles in the development process. Additionally, the interface can accommodate various hand sizes and provides training for tasks such as operating specialized equipment, thereby reducing accidents. These results indicate that the system is well-designed to meet user needs and enhance their experience.
- The Sustainable dimension has an average score of 0.82, indicating moderate effectiveness in promoting sustainability. Factors such as reducing the use of harmful raw materials (0.7) and efficiency of recycling (0.7) suggest room for improvement in these areas. However, the system scores highly in reducing greenhouse gas emissions (0.9), green product design (0.85), reducing energy use (0.85), optimizing material usage (0.9), and promoting the use of recycled materials and reused components (0.85). These scores highlight the system’s strengths in minimizing environmental impact and resource consumption. The proposal includes using low-consumption components, enhancing environmental sustainability, replacing replaceable components to avoid reliance on rigid designs that cannot be replaced, and replacing batteries. However, further efforts could be made to enhance sustainability practices, particularly in sourcing eco-friendly materials and improving recycling efficiency.
- The Sensing dimension stands out with the highest average score of 0.98, reflecting the system’s exceptional performance in this area. Infrastructure investment received a perfect score (1), indicating that the system requires minimal resources to deploy, making it cost-effective and accessible. Maintenance scored 0.95, demonstrating the system’s emphasis on proactive maintenance practices that extend the lifespan of equipment and infrastructure. These results suggest that the system is not only highly effective but also sustainable and economical in its deployment and upkeep. However, it is currently in an experimental phase, limiting its usability in the current version, but with significant potential for progression towards a more natural and intuitive usability.
- The Smart dimension also performs well, with an average score of 0.88. The system scores highly in utility during the use phase (0.9), indicating that it is highly functional and beneficial during its operational phase. Innovation scored 0.9, reflecting the system’s incorporation of advanced features and cutting-edge technology. Performance scored 0.85, showing that the system effectively optimizes worker performance. These high scores in the Smart dimension highlight the system’s advanced capabilities and significant contribution to improving operational efficiency and effectiveness. The investment required for interface development is low relative to its social impact, reflecting its economic value.
- The Safe dimension shares the same average score of 0.88 as the Social and Smart dimensions, indicating strong performance in ensuring quality, durability, health, and safety. Quality and durability received a score of 0.9, reflecting the system’s adherence to high-quality standards and long-term reliability. Health and safety also scored 0.9, demonstrating the system’s effectiveness in improving workplace health and safety and ensuring employee well-being. Quality assurance scored 0.85, indicating robust processes that uphold product standards and customer satisfaction. These results suggest the system is highly reliable and contributes significantly to creating a safe and healthy working environment.

The comprehensive analysis of the questionnaire results has provided significant insights into the effectiveness of the virtual training system incorporating haptic gloves. Participants demonstrated diverse levels of pre-existing knowledge and experience, with notable improvements observed across the training stages. The initial unfamiliarity with haptic gloves did not impede their overall engagement and learning outcomes.

## 4. Discussion

Developing haptic gloves as smart interfaces represents an advancement in assisting operators in industrial settings. This research explored the potential of haptic and virtual environments as innovative tools for enhancing efficiency and safety in manufacturing environments. Thus, the study’s core findings were:

1. Successful communication between the haptic gloves prototype and the virtual platform:
  - Enabled the transfer of hand orientation data.
  - Allowed the representation of movements and rotations from the operator’s hand within the virtual environment.
  - Ensured an accurate and realistic user experience, empowering operators to interact effectively with virtual industrial equipment and process representations.

1. Ergonomic design and usability:
  - This design eased user acceptance and adoption.
  - Validation tests with users indicated that participants perceived haptic gloves as comfortable, user-friendly, and conducive to prolonged use in industrial settings.
  - The positive reception underscores the importance of ergonomic considerations in the design and deployment of smart interfaces for Industry 4.0 applications
    Nevertheless, some challenges and requirements were faced in order to enhance representation:
  - Encountered obstacles such as the need for more realistic hand-object interaction and the development of algorithms for hand movement.
  - Additional physics details are required to enhance representation fidelity in the virtual environment.
  - Current challenges with correctly grasping objects with the virtual hand lead to issues with collisions and affect the expected feedback.
  - Algorithms requiring substantial computational resources for processing 6-degrees-of-freedom IMU data and visualizing the displacement of the virtual hand in the software must be integrated for better hand movement representation.

The study’s findings align with existing literature, such as the works of (Gorecky et al., 2017; Radhakrishnan et al., 2021), which have explored the use of VR for skill enhancement and procedural training. These studies highlight VR’s capability to simulate real-world conditions effectively but also point out the need for more immersive and interactive feedback mechanisms. By integrating haptic gloves that provide tactile feedback, this study advances the field, offering a more immersive training experience that addresses the limitations of previous studies.

Moreover, prior research by (Adattila et al., 2024; Holuša et al., 2023), have demonstrated the effectiveness of VR in industrial training but often lacked comprehensive usability evaluations. Thus, our study addresses this gap by employing a detailed questionnaire to assess the system’s usability across dimensions such as ease of learning, efficiency, and user satisfaction. The high usability scores reflect the system’s effectiveness and user-friendly design, promoting broader adoption of such technologies in industrial settings.

Further supporting the study’s relevance, research by (Müller et al., 2020; Xu et al., 2021) have emphasized the need for human-centric technologies in Industry 5.0, focusing on enhancing worker engagement and productivity. The positive feedback regarding the ergonomic design and intuitive nature of the haptic glove system aligns with these studies, highlighting its potential to enhance worker satisfaction and productivity in line with Industry 5.0 principles.

Finally, applying the 5S principles (Social, Sustainable, Sensing, Smart, Safe) as introduced by (Molina et al., 2024) provided a holistic framework for evaluating the technological implementation. This study applied these principles to assess the impact of the haptic glove and VR system. The high scores in the Social and Safe dimensions reflect the system’s positive impact on worker well-being and safety. However, the lower scores in the Sustainable dimension indicate the need for improvement in reducing environmental impact and enhancing recycling efficiency. This comprehensive evaluation framework offers valuable insights for future developments, ensuring balanced advancements across all dimensions.

Hence, integrating haptic gloves within virtual environments represents a promising advancement in industrial training and operational efficiency. While the study highlights significant achievements, it also points to areas requiring further refinement to fully realize the potential of these technologies in creating safer, more efficient, and user-friendly industrial environments.

Additionally, the development and integration of VR environments for operator training in industrial settings signify a substantial advancement in both workforce development and operational efficiency. VR environments serve as robust platforms for simulating real-world industrial scenarios and delivering hands-on training to operators. Our research findings underscore the effectiveness of VR-based training programs in facilitating experiential learning and skill acquisition. During the evaluation, participants widely regarded the virtual environment as intuitive and easy to comprehend. Additionally, the implementation of haptic gloves enabled users to interact with the virtual milling machine, reducing errors when transitioning to operating the real milling machine. Nonetheless, recommendations were made to enhance the clarity of instructions, such as emphasizing the button sequence for improved understanding.

Concerns persist regarding using VR as a substitute for real-world industrial scenarios due to potential shortcomings in-depth and consequences within virtual environments. These concerns raise ethical dilemmas about trainees’ potential overconfidence. Effective training requires accuracy and authenticity to avoid distorting trainees’ perceptions and compromising workplace safety (Ponce et al., 2024). Misleading representations can erode trust in virtual training programs among stakeholders. Addressing these concerns needs rigorous testing, collaboration with industry professionals, and the creation of additional scenarios. Regular assessment and feedback are vital for identifying areas for improvement and ensuring that virtual training programs adequately prepare individuals for real-world scenarios. Additionally, while this research underscores the benefits of VR-based training in industrial settings, further research is needed to develop advanced VR technologies such as augmented reality and mixed reality to enhance training realism and interactivity.

## 5. Conclusion

This research has demonstrated the potential of haptic gloves integrated with virtual environments to enhance training effectiveness and safety in industrial settings. The findings highlight the successful integration of haptic gloves with virtual platforms, enabling accurate hand orientation data transfer and realistic user interactions with virtual industrial equipment. Additionally, user feedback has validated the gloves’ ergonomic design, indicating high levels of comfort and usability conducive to prolonged use in industrial settings. Despite these advancements, the study also identified several challenges that need to be addressed to enhance the fidelity of hand-object interactions and the accuracy of virtual hand movements. Improvements in algorithms and the inclusion of additional physical details are necessary to provide more realistic feedback and minimize issues related to object grasping and collisions in the virtual environment.

Moreover, integrating VR environments for operator training significantly advances workforce development. Participants found the VR environment intuitive and effective for experiential learning, which was further enhanced by using haptic gloves. However, the study also acknowledges concerns about the potential shortcomings of VR training compared to real-world scenarios, emphasizing the need for continuous assessment and improvement of these virtual training programs to maintain accuracy and authenticity.

By employing a human-centric approach based on the 5S principles—Social, Sustainable, Sensing, Smart, and Safe—this study has shown that advanced digital technologies can significantly improve user experience, operational efficiency, and sustainability in smart factories. Moreover, integrating VR environments for operator training significantly advances workforce development. Participants found the VR environment intuitive and effective for experiential learning, which was further enhanced by using haptic gloves. However, the study also acknowledges concerns about the potential shortcomings of VR training compared to real-world scenarios, emphasizing the need for continuous assessment and improvement of these virtual training programs to maintain accuracy and authenticity.

The primary contributions of this work include:

- Development of a haptic glove that offers realistic feedback through motor vibrations, enhancing user immersion in virtual environments.
- Integration of this glove into a virtual training environment for industrial machinery, significantly reducing real-world operational errors.
- A comprehensive evaluation using the 5S Human-Centric approach, providing insights into the system’s social, sustainable, sensing, smart, and safe dimensions.

The positive feedback from users highlights the system’s usability, ergonomic design, and its potential to minimize accidents by providing effective training before real-world application. The study has also underscored the importance of incorporating user-centered design principles and proactive maintenance practices to extend the lifespan of equipment and infrastructure.

Future research should focus on several key areas to build on these findings. Customization and parametrization are critical in developing haptic gloves in various sizes and materials to cater to different user preferences and physical characteristics, ensuring greater comfort and better fit. Adjustable settings tailored to individual user needs and preferences will enhance the overall user experience. Artificial Intelligence and Machine Learning should be employed to adapt training programs to individual user needs, optimizing learning outcomes and process efficiencies. Besides, tailoring interfaces through these advanced technologies can significantly improve user engagement and effectiveness. Sustainability and environmental impact are also crucial areas of focus.

Incorporating eco-friendly materials and manufacturing processes can further reduce the environmental impact of haptic gloves and associated technologies. Efforts should be made to reduce energy consumption by developing more efficient components or energy-harvesting technologies. Real-world application and scalability need to be explored through extensive field trials in various industrial settings to gather more data on the usability and effectiveness of haptic gloves in real-world scenarios. Ensuring the solution’s scalability for different industrial applications will ensure adaptability to various use cases and environments.

Furthermore, cross-disciplinary collaboration is essential, encouraging collaborations between academia, industry, and government to pool resources and expertise, accelerating the development and implementation of smart and green technologies. Facilitating international knowledge-sharing and capacity-building programs will promote the widespread adoption of these advanced technologies globally. Comprehensive impact assessment through longitudinal studies will help assess the long-term impact of using haptic gloves and virtual environments on worker safety, productivity, and overall well-being. Developing and utilizing broader metrics for evaluating the effectiveness of these technologies will consider not only immediate outcomes but also long-term benefits and potential areas for improvement.

This study demonstrates the promising applications of smart interfaces in industrial settings, particularly for enhancing operator training and interaction with virtual environments. By leveraging these innovative tools, end users can achieve enhanced worker safety, skills development, and overall well-being, thus contributing to achieving the SDGs delineated by the United Nations. Future research should focus on overcoming current challenges and exploring advanced technologies such as augmented reality and mixed reality to improve training realism and interactivity further. Adopting smart interfaces represents a transformative opportunity to enhance workplace conditions, stimulate economic growth, and promote sustainable development within the Industry 4.0 framework, paving the way for a more efficient, innovative, and worker-centric industrial ecosystem.

## Data Availability

The authors have declared no data availability in this manuscript.
